# Towards an understanding of barriers to condom use in rural Benin using the Health Belief Model: A cross sectional survey

**DOI:** 10.1186/1471-2458-5-8

**Published:** 2005-01-21

**Authors:** Sennen H Hounton, Hélène Carabin, Neil J Henderson

**Affiliations:** 1 Department of HIV/AIDS and Reproductive Health, Centre MURAZ, Bobo-Dioulasso, Burkina Faso; 2 Department of Biostatistics and Epidemiology, College of Public Health, University of Oklahoma, Oklahoma City, Oklahoma, USA; 3 Department of Health Promotion Sciences, College of Public Health, University of Oklahoma, Oklahoma City, Oklahoma, USA

## Abstract

**Background:**

HIV/AIDS is the most dramatic epidemic of the century that has claimed over two decade more than 3 million deaths. Sub Saharan Africa is heavily affected and accounts for nearly 70% of all cases. Despite awareness campaigns, prevention measures and more recently promotion of anti viral regimens, the prevalence of cases and deaths is still rising and the prevalence of systematic condom use remains low, especially in rural areas. This study identifies barriers to condom use based on the Health Belief Model (HBM) in Benin, West Africa.

**Methods:**

The study was a cross-sectional survey conducted from June to July 2002. Two hundred fifty one (251) individuals were interviewed using a structured questionnaire adapted from a standardized WHO/GAP questionnaire. A logistic regression was used to identify factors associated with condom use.

**Results:**

In spite of satisfactory knowledge on HIV/AIDS transmission, participants are still at high risk of contracting the infection. Sixty three (63) percents of the interviewees reported being able to recognize infected people, and condom use during the last occasional intercourse was declared by only 36.8% of males and 47.5% of females. Based on the HBM, failure to use condom was related to its perceived lack of efficacy [OR = 9.76 (3.71–30.0)] and perceived quality [OR = 3.61 (1.31–9.91)].

**Conclusions:**

This study identifies perceived efficacy (incomplete protective effect) and perceived utilization-related problem (any reported problem using condoms) as the main barriers to condom use. Hence, preventions strategies based on increasing perceived risk, perceived severity or adequate knowledge about HIV/AIDS may not be sufficient to induce condom use. These data will be useful in designing and improving HIV/AIDS prevention outreach programs in Sub Saharan Africa.

## Background

One of the current challenging tasks faced by health professionals and scientists worldwide is the prevention and control of HIV/AIDS. This disease claims yearly a huge toll of deaths, productivity and economic losses, especially in sub-Saharan Africa where the population is already weakened by poverty, malaria and tuberculosis [1,2]. Curtailing the HIV/AIDS pandemic requires a holistic approach [3]. In Benin, several programmes have been developed to target high-risk groups and to modify cultural risk factors for the transmission of the infection [4-7]. Nonetheless, the prevalence of HIV infection and the rate of other sexually transmissible Infections (STI), and the number of people living with HIV/AIDS (PLWHA) are still increasing [8,9]. In Benin (and in most of West African countries), limited accessibility to anti retroviral medication (ARV) has pushed public health authorities to focus on prevention measures. Nowadays, most of the efforts are being shifted towards access to ARV, care to PLWHA [10], and second generation surveillance. However, there is a scarcity of operational research designed to identify barriers and facilitators to behavioural change [11,12]. This study, which was conducted in rural Benin, identifies factors deterring condom use that could be targeted by HIV outreach programmes using the Health Belief Model (HBM) framework. The Health Belief Model postulates that an individual's actions are based on beliefs. It underlines main factors for decision making such as perceived vulnerability or susceptibility, perceived severity of the outcome or conditions, perceived efficacy or benefit of control measure and the perceived barriers to prevention. It has been extensively used in behavioural sciences to predict behaviours and to design behavioural prevention programs. [13-15]

## Methods

### Study location and participants

Benin is a West-African country with a population of 6.2 million people. The male/female sex ratio is 0.96, 48.5% of the population is under age 15, the rate of literacy is nearly 30.0 % and farming as the main occupation [2]. The study was conducted in Toffo, a county with a population of 80,000 inhabitants, located 50 miles north of Cotonou, the economic capital of Benin. Individuals aged 15 to 55 years old living in Toffo between June 10 and July 23, 2002 were invited to participate in the study. Health professionals, individuals less than 15 years old and school teachers were excluded from the study. Authorization to conduct the study was obtained from the Ministry of Health and the National STI/HIV/AIDS programme in Benin.

### Sample size, sampling and data collection

The sample size was calculated based on results from the Benin 2001 Demographic and Health Survey [2]. Using the proportion of condom use reported in that survey (14.6%) and to detect a difference of 10% with a power of 90% and an error of 5%, a sample size of 245 people was needed [16]. A stratified random sample was applied using the 10 villages of Toffo as strata, and an average of 25 individuals per stratum was invited to participate after a brief presentation of the study, its aims and its potential applications. Participation was voluntary and participants could withdraw anytime during the interview. 270 individuals in the catchments area were invited and 251 accepted to participate in the study. Sixteen completed forms were removed from the analysis because of incomplete data on demographic characteristics and key responses related to the Health Belief Model. The final sample size was 235. Data were collected using a questionnaire adapted from the surveillance questionnaire developed by the Global AIDS Program of the World Health Organization (see survey items in Annexes). A pretest of the questionnaire was carried out on a convenient sample of 20 people of both genders living in Toffo and interviewers (3 social workers, one female and 2 males) were trained by SHH using simulated interviewees.

### Data analysis

Data were stored in a file using Epi info 2000 [3] and then imported in SPSS 11.0 [17] and in SAS 8.1 [18]. The analysis included descriptive statistics, cross-tabulation and logistic regression. The following variables were included in the analysis: socio-demographic characteristics, perceived vulnerability (participant feeling at risk or not), perceived severity of the disease (AIDS perceived as deadly or not), perceived efficacy (condom effective to prevent infection or not), perceived barrier (any reported problem with use of condoms) and condom use during the last occasional sexual intercourse

## Results

### Socio-demographic characteristic

The age distribution of the study population was similar to that of the Benin population [2]. Table 1 shows that there were 1.9 times more males than females sampled and the former were older (difference between mean age = 3.2 years with 95%CI = 1.8–4.5). A proportion of 69% of the participants declared being married (monogamous or polygamous) and 28% single. Farming was the most common reported occupation (37.4%) followed by laborers and small business. Sixty-six percent of males compared to 30% of females declared not having had any school education. The most common reported religion was Christianity (52 %) albeit polytheism being a social fact in Benin. Nearly two-thirds of the participants (63%) declared "Fon" as their ethnic group. There was no significant difference in socio-demographic variables by gender.

**Table 1:** Socio-demographic characteristics of 235 participants in a HIV/AIDS interview, Toffo county, Benin (June–July 2002)

Characteristics	Males N (%)	Females N (%)	Total N (%)
**All participants**	155 (66.0)	80 (34.0)	235
**Age groups**			
15–24	56 (36.1)	37 (46.3)	93 (39.6)
25–34	52 (33.5)	29 (36.3)	81 (34.4)
35–44	30 (19.4)	5 (6.3)	35 (14.9)
45 +	17 (11.0)	9 (11.3)	26 (11.1)
**Marital status**			
Married, monogamous	87 (56.1)	27 (33.8)	114 (48.5)
Married, polygamous	23 (14.8)	24 (30.0)	47 (20.0)
Single	44 (28.4)	21 (26.3)	65 (27.7)
Widow, Separated	1 (0.7)	8 (0.9)	09 (07.3)
**Occupation**			
Farming	81 (52.3)	7 (8.8)	88 (37.4)
Working class	48 (31.0)	7 (8.8)	55 (23.4)
Small business	10 (6.5)	33 (41.3)	43 (18.3)
Housewife	NA	21 (26.3)	21 (08.9)
High school student	4 (1.7)	11 (4.7)	15 (06.4)
Other	12 (7.7)	1 (1.3)	13 (05.6)
**Educational level**			
None	47 (30.3)	48 (60.0)	95 (40.4)
Some	108 (69.7)	32 (40.0)	140 (59.6)
**Ethnicity**			
Fon	104 (67.1)	46 (57.5)	149 (65.1)
Aizo	26 (16.8)	24 (30.0)	49 (21.4)
Other	25 (16.1)	10 (12.5)	37 (13.5)
**Religion**			
Christian	42 (52.5)	80 (51.6)	122 (51.9)
Muslim	3 (3.8)	2 (1.3)	5 (2.1)
Traditional	21 (26.3)	54 (34.8)	75 (31.9)
Other	14 (17.5)	19 (12.3)	33 (14.0)

### Knowledge

Table 2 presents a summary of the knowledge of the participants on HIV/AIDS and crude odds ratio estimates comparing males to females. There was a high awareness of AIDS (99, 9%), and its perceived risk (97% of participants considered AIDS as a deadly disease) among participants. Females were more aware than males of the modes of transmission of HIV infection (87 % of females versus 50% reported knowing at least 2 modes of transmission). There was a difference in preventive measures for HIV/AIDS by gender: Females reported mainly fidelity and abstinence whilst males primarily reported condom use. In addition, 84% of females whereas 52% of males reported being able to identify an HIV-infected person. This indicates the need for improving knowledge of the disease in general for both genders. Education level and religion did not have a meaningful effect on knowledge across age groups.

**Table 2:** Distribution of knowledge by gender of 235 participants in a HIV/AIDS interview, Toffo county, Benin (June–July 2002).

**Knowledge**		N (%)	Difference* (%)
	Male	Female	[95% CI]
Have you ever heard of HIV/AIDS?			
Yes	154 (99.4)	80 (100)	- 0.6 [-1.8; 6.2]
What is your source of information?			
Radio (± other sources)	146 (94.2)	58 (72.5)	21.7 [11.3; 32.2]
Health professionals (only)	4 (2.6)	8 (10.0)	- 7.4 [-14.4; -0.4]
Friends (only)	5 (3.2)	14 (17.5)	-14.3 [-23.1; -5.5]
According to your knowledge, what is AIDS?			
Deadly disease	134 (86.5)	79 (99.8)	-13.3 [-18.7; -7.8]
Projected disease	17 (11.0)	1 (1.2)	9.8 [4.3; 15.3]
Don't know	3 (1.9)	0 (0.0)	1.9 [-2.5; 4.0]
Other	1 (0.6)	0 (0.0)	0.6 [-0.6; 1.8]
Modes of transmission of HIV?			
Knows at least two modes of transmission	77 (49.0)	70 (87.0)	-38 [-48.8; -27.2]
Knows sexual transmission	68 (43.9)	6 (7.5)	36.4 [26.7; 46.1]
Do not know any	9 (6.5)	4 (5.0)	1.5 [-4.6; 7.6]

**Prevention**			
			

What are the prevention methods of getting HIV?			
Abstinence /fidelity	13 (8.4)	44 (55.0)	-46.6 [-58.3; -34.8]
Serological test.	0 (0.0)	8 (10.0)	-10 [-16.6; -3.4]
Condom	131 (84.5)	20 (25.0)	59.5 [48.4; 70.6]
Don't know	4 (2.6)	0 (0.0)	2.6 [0.0; 5.1]
Other	7 (4.5)	8 (10.0)	-5.5 [-12.8;1.8]
How can a HIV-infected person be identified?			
Symptoms	80 (51.6)	67 (83.8)	-32.2 [-43.5; -21]
Can not differentiate	23 (14.8)	2 (2.4)	12.4 [5.9; 19]
Don't know	49 (31.6)	11 (13.8)	17.8 [7.3; 28.3]
Other	3 (2.0)	0 (0.0)	2 [-0.2; 4.2]

Difference* = Difference between males and females in the prevalence of knowledge with 95 % confidence interval (using a binomial distribution)

### Behavioural risk factors

The overall condom use in this population was low (34%). Table 3 describes the distribution of frequency of not ever using condom, last occasional intercourse without condom use and median number of sexual partners during the past 12 months, according to age, gender, education and marital status. Single participants with some education declared using condom more frequently. The proportion of subjects who declared using condom decreased with age and with males being marginally more likely to declare using it than females. We did not find any significant difference about reports on the use of condom during the last occasional intercourse by age groups, gender, educational or marital status. The small number of individuals declaring condom use during the last occasional intercourse maybe the reason why no significant associations were found.

**Table 3:** Distribution of selected behavioural risk factors of 235 participants in a HIV/AIDS interview by age groups, gender, education level and marital status in Toffo county, Benin (June–July 2002)

Behavioural risk factors	Number of participants who declared not using condom **N (%)**	Last occasional intercourse without using condom **N (%)**	Median number of sexual partners during the last 12 months **N (%)**
**Age group**			
15–24	56 (60.2)	54 (58.1)	1.0
25–34	50 (61.7)	49 (60.5)	1.0
35–45	26 (74.3)	23 (65.7)	1.0
45 +	22 (84.6)	14 (53.8)	1.0
**Gender**			
Male	96 (61.9)	98 (63.2)	2.0
Female	58 (72.5)	42 (52.5)	1.0
DIFF [95% CI] Female/Male	10.6 (-2.0; 23.0)	- 10.7 (-24.0; 3.0)	NA
**Education**			
None	73 (76.8)	54 (56.8)	1.0
Some	81 (51.9)	86 (61.4)	2.0
DIFF [95% CI] None/Some	24.9 (13.0; 36.8)	- 4.6 (-17; 8.0)	NA
**Marital status**			
Single	36 (55.4)	34 (52.3)	2.0
Other	118 (69.4)	106 (62.4)	1.0
DIFF [95%CI] Other/Single	14 (.07; 28)	10.1 (-4; 24)	NA

**DIFF **[95% CI] = Difference in Proportion with 95 % confidence interval

NA: Not Applicable

However, it is interesting to note that whilst females declared ever using condom less often than males, they declared having used the condom during the last occasional intercourse more often. In particular, even though 73% of females declared never using the condom, 47% declared having used it during the last occasional intercourse. None of these differences were significant but they clearly indicated contradictory tendencies and answers to the questionnaire. It may be that among females who ever used condom, they use it more frequently than males. Finally, the median number of sexual partners during the last 12 months varied by gender (2 for males versus 1 for females), by education (2 for some versus 1 for none) and by marital status (2 for single versus 1 for other) but not by age groups.

### Determinants of condom use behavioural change

Overall, there was a *high perceived risk *of contracting HIV infection among interviewees: 94% considered themselves as vulnerable to HIV/AIDS. This proportion was higher in females compared to males. Similarly there was a *high perceived severity *of HIV/AIDS: 99% of females compared to 87% of males perceived HIV/AIDS as a severe and deadly disease. Conversely, there was a relatively *low perceived efficacy *of condom as a protective measure: only 37% of the interviewees perceived condom as an effective mean in protecting from getting HIV infection. We identified several socio-cultural *barriers *to behavioural change namely reported problems using condom (88% of the interviewees), the alleged capability to physically recognize an HIV infected person and the denial all together of the disease (only 19% participants believe HIV/AIDS exists). Also, cultural practices such as polygamy (20% of the study population), poverty, the belief that there is a cure for the disease (74%) and religion (9 % of non favorable reaction towards condom are among declared Christians) were all not favorable to HIV infection control.

### Logistic regression using the theoretical health belief model

Table 4 describes the results of a logistic regression, fitted to assess the strength of association between perceived vulnerability (participant feeling at risk or not), perceived severity of the disease (AIDS perceived as deadly or not), perceived efficacy (condom effective to prevent infection or not), perceived barrier (problems with using condoms) and the lack of condom use. Perceiving condom as ineffective (OR = 9.8, 95%CI = 3.2–30.0) and having reported problems with using the condom (OR = 3.6, 95%CI = 1.3–9.9) were both associated with the lack of use of condom. However, perceiving oneself as vulnerable to HIV infection (OR = 6.9, 95%CI = 0.9 – 52.5) was not strictly statistically significant since most interviewees felt vulnerable, reducing the power of detecting a significant difference. This variable was also a weak confounder for the effect of perceiving the condom as ineffective. Not perceiving HIV/AIDS as a deadly disease (OR = 2.5, 95%CI = 0.3 – 19.7) was not associated with the lack of use of condoms.

**Table 4:** Crude* and adjusted** odds ratio (OR) estimates with their 95% Confidence Interval (95% CI) of the effect of perceived efficacy of condom, barriers to condom use, vulnerability and severity on the lack of condom use for 235 participants in a HIV/AIDS interview, Toffo county, Benin (June–July 2002).

Variables	Crude OR^1 ^(95% CI)	Adjusted OR^2 ^(95% CI)
**No perceived risk to HIV infection**	6.9 (0.9 – 52.5)	NA^3^
**AIDS not perceived as a deadly disease**	2.5 (0.3 – 19.7)	NA^3^
**Perceived incomplete protection using condoms**	11.5 (3.8 – 34.7)	9.8 (3.2 – 30.0)
**Reporting any problem using condom**	5.4 (2.1 – 13.7)	3.6 (1.3 – 9.9)

2 LL Chi-square: 32.2 P-value < 0.0001

Score Chi-square: 32.2 P-value < 0.0001

C-statistic = 0.81

^1^Crude OR: calculated from a univariate logistic regression

^2 ^Adjusted OR: Calculated from a multivariate logistic regression including Perceived incomplete rotection using condoms and reporting any problem using condom.

^3 ^NA: Not Applicable because these variables did not have an important effect on the use of condom.

## Discussion

This study was the first ever to use the Health Belief Model (HBM) to assess cultural behaviour in rural Benin towards condom use and HIV/AIDS. The HBM was reported to be one of the most widely used behavioural frameworks for more than five decades but has been criticized for its inability to efficiently predict people's behaviour [14]. There is general agreement that the components of HBM should include self-efficacy and cues to action, and that susceptibility and severity should be conditional on action or inaction [13,14]. The lack of generally accepted model construct also makes comparisons difficult across studies. An effort was made in this study to address these concerns by clearly defining the model's construction.

Our results showed there is a high awareness on AIDS in general and that women knew more about the modes of transmission of HIV/AIDS and its impacts than men. Conversely, women were more likely to feel that they could identify HIV-infected individuals from their symptoms. In addition, females were less likely to declare using condom in general even though a higher proportion declared having used condom during the last occasional sexual intercourse. This finding is disturbing and could be explained by the difference in perception of the question "do you use condoms?" It is difficult to judge what the true answer is but it is likely that rare events are better reported, and thus women may be more prone to recall the use of condom than men during occasional intercourse given that they declared on average fewer sexual partners. It is also possible that among women who do use condom, they will use it more regularly than men.

Our measure of perceived vulnerability might not be sensitive enough to capture differences in perceived risks. In fact, all women and most men felt they were at risk of acquiring the infection, yet only a small proportion were using condoms. Another explanation may be that perceived risk is not a driving force in behavioural change in this subset of the population. This is an illustration of the complexity of modeling human behaviour and can thus make a case for further cultural-specific HIV-behavioural research. When only considering the percentage of condom use by gender, females appear to be at a higher risk of acquiring HIV even though they appeared to know more about transmission routes and prevention methods. This might be due to the well established difficulty facing women in negotiating the terms of sexual intercourse. In fact, gender inequality is associated with poverty, condom with distrust and sexual economic exchange is not perceived as prostitution [19]. All these factors make women vulnerable to acquire HIV infection, and therefore it is important to consider empowerment of women, gender inequality and poverty as key strategies of HIV/AIDS prevention programmes.

Despite a relatively acceptable knowledge of modes of transmission and prevention methods, only a few of participants declared using condoms, which is an indication that a relatively good knowledge about HIV/AIDS, even though necessary, may not be a key factor in behavioural change in fighting HIV epidemic in the study population. These findings also indicate that programmes which aim only at increasing awareness and knowledge may not succeed.

Using the HBM to analyze the determinants of behavioural change in our study population, we can conclude that there is a high-perceived vulnerability and perceived severity, and yet this does not encourage condom use. An important proportion of participants do not believe in the efficacy of condoms and there are barriers to the use of condoms.

Our results are comparable to that found in a similar study in the USA [20] and in a review of published studies using HBM [14] where perceived barriers were found to be the single most powerful predictors of the HBM. Our findings are also consistent with results of studies conducted in Kenya [21] and in Ghana [22], in which perceived barriers were found as being the strongest predictors of condom use. However, these results can not be generalized across settings For example, in a study conducted among American university students, the HBM did not significantly explain condom use but rather condom use was associated with sexual practices [23]. Perceived benefit of avoidance of pregnancy was found as one of the strongest predictors of consistent condom use in New York female adolescents [24] and in Zimbabwe social support was found to be the most consistent factor associated with sexual risk reduction [25]. These observed differences in the strongest(s) predictor(s) of the HBM can be noticed through several other works [26-30]. Hence, it appears important to conduct operational behavioural researches in each local setting to identify factors that influence condom use.

One limitation of our study was that for ethical reasons, subjects less than 15 years old were excluded even though some may have already been sexually active. Also, there was a potential selection bias by not having equal number of interviewers by gender, which resulted in an over-sampling of males. Our results would be biased if the reason for poor recruitment of women was linked to their behaviours, which is not likely to be the case. There were three males interviewers for one female (difficulties in recruiting female educated social worker in the area), and interviewer/participants must be from same gender. For the purposes of the analysis we assumed that reported knowledge and behavioural risk factors are independent. Finally there is no evidence for the validity or reliability for the original WHO questionnaire, however its use allows for comparability of results across settings.

## Conclusions

Condom use, in our study population, depends on its perceived quality and perceived efficacy. There is an indication that behavioral communication change strategies based on increasing perceived risk or vulnerability of the population or based on fear factor by increasing perceived severity of HIV/AIDS are less likely to be deterrent towards condom use and require more researches. HIV outreach programs must target more barriers of condoms use. Condom outreach programmes should be defined at community level and must be defined in association with the community, using problem-solving techniques and selecting the most relevant targets, based on their importance and changeability [13]. Data from this study could be useful for the design and planning of health intervention programmes, resource allocation and evaluation of condom outreach activities in Benin.

## Competing interests

The author(s) declare that they have no competing interests.

## Authors' contributions

SHH conceived of the study, designed the protocol, carried out and supervised the field work and data collection, performed and interpreted the statistical analysis and wrote the manuscript. HC participated in analysis and interpretation of the data, and in the writing of the manuscript. NJH contributed in earlier analysis of the data and reviewed the manuscript. All authors read and approved the final manuscript.

**Table 5:** Survey items, HIV/AIDS and condom use survey, Toffo county, Benin (June – July 2002).

**SURVEY ITEMS**	**RESPONSE CATEGORIES**
**General knowledge on HIV/AIDS**	
1. Have you ever heard about HIV/AIDS	Yes/No
2. In your knowledge how severe is HIV/AIDS	Deadly, don't know, imaginary, other
3. How could someone get infected by HIV?	At least 2, one or no correct answer(s),
4. Who you think are at risk of getting HIV?	Everyone/specific groups/Don't know
5. In your knowledge, what are the prevention methods of getting HIV?	Abstinence, Fidelity, Condom, Other, Don't know
6. How could you recognize a HIV-infected person?	Could not, Cachexia, Other symptoms, Don't know
**Beliefs on HIV/AIDS**	
7. Do you believe HIV really exists?	Yes/No/Don't know
8. Do you think you are at risk of getting HIV?	Yes/No/Don't know
9. If no to 8) why?	Fidelity/Condom use/Other/Don't know
10. Where you believe HIV originates from?	God/Bewitchment/Other/Don't know
11. Do you think one can completely cure from HIV/AIDS?	Yes/No/Don't know
12. If yes to 11) how?	Medicine/Herbs/Prayers/Other/Don't know
13. How would you rate the protective effect provided by condoms?	Complete/Incomplete/Useless/Don't know
14. Does your religion believe HIV exists?	Yes/No/Don't know
15. What is the position of your religion towards condom use?	Favorable/Unfavorable/Indifferent/Don't know
	
**Behaviors and attitudes**	
16. Would you mind taking a HIV screening test if you were asked?	Yes / No / Don't know
17. Do you use condoms?	Yes / No / No answer
18. If No or No answer to 17) why?	Don't like / Only God save / Other
19. How often do you use condoms?	Always / Sometimes / Never
20. Did you use condom during the last occasional intercourse?	Yes / No
21. Do you encounter any problem using condoms?	Yes / No
22. If yes to 21) what type of problems	Less lubrificated / Less pleasure / break easily / Other
23. Numbers of sexual partners during the last 12 months	
	
**Socio demographic characteristics**	
24. Age	Full years
25. Sex	Male / Female
26. Marital status	Married monogamous / Married polygamous/ Single / Divorced / Widowed / Separated
27. Education (Ability to read)	Fluent / With difficulty / Not able to read at all
28. Occupation	Student / Farmer /
29. Religion	Christianity / Islam / Animist / Other
30. Place (please give the name of your village)	Village name

Adapted from the World Health Organization / Global AIDS Program's questionnaire

## Pre-publication history

The pre-publication history for this paper can be accessed here:


